# Depression, Anxiety and Eating Disorder-Related Impairment: Moderators in Female Adolescents and Young Adults

**DOI:** 10.3390/ijerph18052779

**Published:** 2021-03-09

**Authors:** Johanna Sander, Markus Moessner, Stephanie Bauer

**Affiliations:** Center for Psychotherapy Research, Heidelberg University Hospital, 69115 Heidelberg, Germany; markus.moessner@med.uni-heidelberg.de (M.M.); stephanie.bauer@med.uni-heidelberg.de (S.B.)

**Keywords:** eating disorders, depression, anxiety, adolescence, perfectionism, self-esteem, mood, emotion regulation

## Abstract

Adolescents and young adults, particularly females, are highly vulnerable to the development of anxiety disorders, depression, and eating disorders. Comorbid anxiety disorder or depression in eating disorders are associated with greater symptom severity, poorer prognosis, and burden of illness. Nonetheless, studies on what affects the relationship between anxiety, depression, and eating disorders in female at-risk samples are scarce. Using hierarchical linear modeling, the present study examined potential moderators to explain between-person differences in the association between anxiety, depression, and eating disorder-related impairment within 12- to 25-year-old females (*N* = 320). High impairment in anxiety/depression was associated with more severe eating disorder symptoms. Older age as well as greater impairment in mood dysregulation, self-esteem, and perfectionism were linked to more severe eating disorder symptomatology. Whereas mood dysregulation, self-esteem, and perfectionism had no statistically significant moderating effects, younger age appeared to augment the association of anxiety/depression and eating disorder symptomatology. Preventive care in particular needs to consider age-related effects as eating disorder symptoms are associated more strongly with symptoms of anxiety and depression in early adolescence.

## 1. Introduction

Adolescence is a phase of profound socioaffective and neurocognitive changes reaching into early adulthood which is accompanied by a heightened vulnerability for onset of mental problems and disorders [1,2]. Female adolescents and young adults are particularly at risk for eating disorders [3,4]. In a large Austrian sample, 31% of female adolescents aged 10 to 18 years were found to be at risk to develop an eating disorder [5]. In young women, eating disorder symptoms are highly prevalent. Ward and colleagues estimated the disease dynamics of eating disorders up to the age of 40 based on nationally representative US survey data and found the highest annual prevalence at age 21, with initial onset mostly before the age of 25 [6]. High vulnerability for eating disorders in this age group is marked by specific risk factors such as body dissatisfaction and body image concerns.

Anxiety disorders and depression are among the most prevalent mental disorders in adolescence [7]. Both are the most common comorbid diagnoses in eating disorders [8,9], especially in adolescence [10]. In a recent study on 15- to 25-year-old females, those with lifetime major depressive disorder or anxiety disorder were four times more likely to have a lifetime eating disorder [11].

Similar to anxiety disorders, individuals with an eating disorder use dysfunctional strategies such as disordered eating to cope with their emotions [12]. In addition, persisting comorbid anxiety symptoms after eating disorder remission increase risk of relapse [13] as individuals revert to former eating disorder-related coping behaviors [14]. In female adolescents and young adults, comorbid anxiety disorders are associated with greater eating disorder psychopathology [15]. Specifically, comorbid social anxiety hampers recovery efforts as avoidance of interpersonal situations and fears of negative evaluations interfere with treatment engagement and building a good therapeutic relationship [16].

Increasing prevalence rates are reported for depressive disorders in adolescents and young adults, e.g., in the US [17], the UK [18], and Finland [19], especially in girls [20]. Concerns about and negative perception of oneself as well as one’s appearance are associated with onset and maintenance of eating disorders and depression [21,22]. Poor therapeutic outcome may be explained by symptoms that reinforce each other: dysfunctional social interaction patterns in depression lead to negative interpersonal experiences that increase the risk for dysfunctional emotion regulation (i.e., disordered eating) [23]. In line with this, eating pathology aggravates with greater severity of affective symptoms [24,25], and depressed mood improves with weight restoration [26].

In summary, evidence supports a strong relationship between anxiety, depression, and eating disorders. Comorbid depression and anxiety symptoms in eating disorders are a marker of greater symptom severity and poorer prognosis and outcome, particularly in young females. To date, only a few studies have explored the differences between individuals with clinical eating disorders with and without comorbid depression and anxiety disorders, e.g., [27,28,29]. A more recent study identified factors such as a restrictive eating disorder subtype and a deterioration of the general health status relative to one year ago as well as medical comorbidities and low educational level as critical causes for anxiety and depression in adult outpatient eating disorder patients [30]. Nevertheless, studies with adolescent samples exploring the link between anxiety, depression, and eating disorder symptomatology going beyond demographic or clinical characteristics (e.g., age at onset, bingeing, and vomiting frequency) are scarce.

Theoretical models that have received the most empirical support are based on the assumption that anxiety, depression, and eating disorders are characterized by a shared etiology [12]. In the present study, the focus is on three aspects that are associated with eating disorders and play a role in the development and maintenance of anxiety disorders and depression. To be more precise, the present study investigates whether these factors moderate the relation between eating disorders and anxiety/depression.

Emotion regulation describes the awareness and recognition as well as the attempt to regulate emotional states in accordance with regulation-related goals [31,32]. Evidence suggests that dysfunctional regulation serves as a transdiagnostic risk and maintenance factor involved in several mental disorders including anxiety, depression, and eating disorders [31]. Maladaptive regulation strategies (e.g., avoidance) are associated with more severe psychopathology in all three disorders [31,33]. It is suggested that disordered eating behaviors themselves are maladaptive strategies to regulate negative emotional states [31]. For instance, higher levels of anxiety and depression may lead to the use of dysfunctional emotion regulation strategies such as disordered eating (e.g., bingeing), possibly resulting in greater eating disorder psychopathology and vice versa. As experience of stress and negative emotions heightens, adolescents and young adults are in greater need of effective emotion regulation [34], and a dysfunctional emotion regulation may be particularly problematic in this age group [35].

In the transdiagnostic theory of eating disorders, low self-esteem and perfectionism are key maintaining factors [36]. The former describes a pervasive negative view, concepts, and beliefs about one’s self-worth [36]. Low self-esteem is linked to negative perception of one’s body and body dissatisfaction, i.e., prominent risk factors for eating disorders [37]. In addition, eating disorder symptoms aggravate low self-esteem as well as negative affect and negative social evaluation (e.g., fear of negative evaluations by others) [38]. Particularly in girls, low self-esteem was found during adolescence and increasing with age [39,40]. Low self-esteem affects a range of health outcomes [41] and presents a prospective risk factor for the development of depression in adolescents and young adults [37,42,43]. Low self-esteem is also characteristic of patients with anxiety disorder [44,45].

Perfectionism is a multidimensional concept encompassing the pursuit of high standards and critical self-evaluation when failing to achieve these standards [46]. According to Fairburn et al.’s transdiagnostic theory, perfectionism is another key maintaining factor for eating disorders [36]. Perfectionism significantly predicted eating disorder-related impairment and was associated with eating pathology [47,48]. In addition, elevated rates of perfectionism are found in anxiety disorders and depression, and it is significantly associated with anxiety and depression in both adolescents with clinical and subclinical eating disorders [49,50,51].

However, the role that dysfunctions in regulating one’s emotions, self-esteem, and perfectionism may play in the association between anxiety, depression, and eating disorder-related impairment and symptomatology is yet to be determined.

### 1.1. Study Aim

Given the high prevalence rates of anxiety, depression, and eating disorders, their high comorbidities, as well as the immense impairment associated with these illnesses, there is a clear need to understand their association more precisely, particularly in young females.

The present study explores the association between anxiety/depression and eating disorder-related impairment in female adolescents and young adults. Based on a large longitudinal dataset, the importance of evidence-supported risk and transdiagnostic factors of eating disorders as potential moderators for this association is investigated.

It was hypothesized that (a) higher levels of anxiety/depression are associated with greater eating disorder-related impairment, and that (b) the strength of this association differs significantly between participants. Finally, it was hypothesized that (c) these differences can be partly explained by four moderators, i.e., mood dysregulation, self-esteem, perfectionism, and age.

### 1.2. Contribution

To date, studies on the moderation of depression, anxiety, and eating disorder-related impairment are scarce. This study contributes to the understanding of transdiagnostic risk factors and etiology as well as maintenance of eating disorders by examining potential moderators longitudinally in a large sample of young females.

## 2. Materials and Methods

### 2.1. Participants and Procedures

Data were obtained from the ProYouth initiative which aimed at the dissemination of an Internet-based eating disorder prevention program (for a description of the ProYouth program see: [52]) into routine care. The ProYouth initiative did not aim at testing the intervention’s efficacy, but rather at investigating different implementation and dissemination strategies. In order to participate in ProYouth, participants completed an initial online screening including questions that assessed demographics, utilization of eating disorder treatment, as well as eating disorder-related risk factors and symptoms. Inclusion criteria were sufficient German language skills and Internet access. Participants endorsing risk for development of an eating disorder in the initial online screening were encouraged to register for the program. Following their registration, they were invited to brief bi-weekly monitoring assessments of eating disorder symptoms and the PHQ-4 (anxiety/depression [53]).

Participants over 25 years were excluded from the analyses. In total, 320 female participants who completed at least two of these bi-weekly assessments were included in the current study. Assessments were conducted using the ASMO software (Center for Psychotherapy Research, Heidelberg University Hospital, Heidelberg, Germany [54]). The mean number of online assessments completed by each participant was 6.64 (*SD* = 9.13); 40.3% of participants completed five or more assessments.

### 2.2. Measures

#### 2.2.1. Initial Online Screening

Within the initial online screening, participants reported their age, sex, body weight and height, of which the latter were used to calculate participants’ body mass index (BMI). Eating disorder-related risk factors were assessed with the Weight Concerns Scale (WCS [55]), the Short Evaluation of Eating Disorders (SEED [56]), and the Eating Disorder Examination-Questionnaire (EDE-Q [57]).

The WCS is a five-item self-report questionnaire on weight and shape concerns on a scale ranging from 0 to 100. A WCS score larger than 57 marks elevated risk of developing an eating disorder. The WCS showed adequate internal consistency in previous studies (*α* = 0.76–0.85 [58,59,60,61]) and good retest reliability and good predictive validity [55,62]. In the present sample, its reliability was excellent (*α* = 0.90).

The SEED was applied to measure BMI and frequencies of behavioral eating disorder symptoms such as vomiting, use of laxative, dieting, excessive exercise, and bingeing in the past week. Frequencies are rated from zero (not at all) to three (several times a day). Construct validity was supported as well as good criterion-related validity, discriminative power, and concurrent validity [56]. In the present study, no scales were calculated, but single items were used to assess BMI and frequencies of compensatory behaviors (i.e., bingeing, vomiting, laxative use, dieting, and excessive exercising) in a validated manner.

The EDE-Q consists of four subscales assessing restraint, eating concern, weight concern, and shape concern as well as global eating disorder psychopathology score. In the present study, the EDE-Q global score was used to assess overall eating disorder severity. The EDE-Q shows excellent internal consistency of α = 0.97 for the global score as well as adequate retest reliability [63,64,65]. In the present sample, Cronbach’s alpha indicated excellent reliability (α = 0.97).

#### 2.2.2. Eating Disorder Symptomatology

Eating disorder symptoms were assessed as part of the bi-weekly monitoring. The monitoring scale consists of 8 items covering body dissatisfaction, impact of eating, weight, and shape on one’s general wellbeing as well as restraint eating, dieting, bingeing, and compensatory measures (i.e., laxative use, vomiting, excessive physical activity) in the past 7 days. Items are rated on a 4-point Likert scale, with high scores indicating greater impairment.

As the monitoring assessment was self-developed, we investigated its psychometric properties for the purpose of this study. Principal component analysis (PCA) was conducted. Kaiser–Meyer–Olkin measure verified the sampling adequacy for the analysis (*KMO* = 0.89). Bartlett’s test of sphericity (*χ*^2^(28) = 10309.89, *p* < 0.001) indicated that correlations between items were sufficiently large for PCA. The analysis revealed one component with an eigenvalue over Kaiser’s criterion of 1, explaining 59.42% of the variance. The scale correlates highly with the Eating Disorder Examination-Questionnaire (EDE-Q [57]) (*r* = 0.772, *p* < 0.001) and the Clinical and Research Inventory for Eating Disorders (CR-EAT [66]) global score (*r* = 0.709, *p* < 0.001), particularly with its subscales eating behavior disturbances (*r* = 0.736, *p* < 0.001) and affective cognitive impairment (*r* = 0.702, *p* < 0.001). In the present study, Cronbach’s alpha indicated high reliability (α = 0.90).

#### 2.2.3. Anxiety and Depression

The Patient Health Questionnaire-4 (PHQ-4 [53]) is a 4-item measure that allows a brief and accurate measurement of core depression and anxiety symptoms. The total PHQ-4 score is an overall measure of symptom burden, functional impairment, and disability. Items are rated on a 4-point Likert scale, with higher scores indicating greater impairment. The cut-off score of ≥9 of the total PHQ-4 score indicates presence of anxiety disorder or depression, and a mean score of ≥5 on the subscales indicates probable cases of anxiety disorder and/or depression. The PHQ-4 has a good internal consistency (*α* = 0.85) and, in the current sample, demonstrated an excellent Cronbach’s alpha of *α* = 0.93.

#### 2.2.4. Mood Dysregulation, Self-Esteem, and Perfectionism

Subscales of the Clinical and Research Inventory for Eating Disorders (CR-EAT [66]) were applied to assess mood dysregulation, self-esteem, and perfectionism. The mood dysregulation scale measures emotionality and emotion processing with eight items, such as, “I simply feel bad” and “Sometimes I am overwhelmed by strong feelings and I don’t know why”. Seven items form the self-esteem scale, including items such as “I think I am just as valuable as other people” and “I am proud of myself”. The perfectionism scale consists of seven items and is measured using items such as, “In my family only outstanding achievements matter” and “Either I do things perfectly or I don’t do them at all”.

The CR-EAT was developed and validated specifically for the use in online assessment of eating disorders. Participants completed the CR-EAT once in the beginning of their study participation. Item responses range from 1 (totally false) to 6 (absolutely correct), and higher scores indicate greater impairment. The CR-EAT demonstrated medium to excellent internal consistency (global score *α* = 0.97; current sample *α* = 0.85). In the present sample, reliabilities for the mood dysregulation, self-esteem, and perfectionism scales are *α* = 0.93, *α* = 0.94, and *α* = 0.82, respectively.

### 2.3. Data Analyses

In order to examine moderation effects, HLM (maximum likelihood) was applied. HLM accounts for the hierarchical data structure and allows modeling moderation effects for intraindividual associations of PHQ-4 and eating disorder-related impairment in accordance with the main focus of this study. In addition, HLM can handle the differences in the number of data points between subjects in this longitudinal dataset. The dependent variable was eating disorder symptomatology. PHQ-4 scores were person-mean centered. Potential moderators were tested by cross-level interactions with the person-mean-centered PHQ-4. The models included the PHQ-4 person mean, random intercepts, and random effects for the person-mean-centered PHQ-4. Moderator effects were tested for age, mood dysregulation, self-esteem, and perfectionism. All analyses were conducted in SPSS version 26 [67].

## 3. Results

### 3.1. Descriptive Data

Sample characteristics are shown in Table 1. The mean age was 16.98 years (SD = 3.61), ranging from 12 to 25 years. The BMI scores of almost 30% of the participants indicated underweight (BMI < 18.5), and the BMI of 62.5% of the participants was in the healthy weight range (18.5 ≤ BMI ≤ 24.9). Half of the participants showed severe weight concerns (WCS > 57 [68]), and almost one third demonstrated frequent fear of gaining weight. In total, 15.63% had been treated for an eating disorder in the past; 25% of the participants endorsed vomiting and about half reported dieting and excessive exercising as compensatory measures for fear of weight gain. More than half reported binge-eating at least once in the past week. Given these characteristics, the sample may be overall described as a young female sample displaying a risk for or symptoms of eating disorders.

Evidence of elevated anxiety/depression levels was found in 18.4%. Regarding the PHQ-4 subscales, 16.6% indicated probable presence of anxiety disorder and 12.5% of depression. Participants reported elevated mood dysregulation and perfectionism as well as rather low self-esteem.

### 3.2. Eating Disorder Symptomatology and Anxiety/Depression

Intraclass correlation (ICC) shows that 81.05% of the variance in eating disorder symptoms was due to differences on the between-person level, i.e., over time, eating disorder symptoms varied to a greater extent between participants than they did within-person. Model 1 (Table 2) shows the parameter estimates from the random coefficient model. Results indicate a positive association between PHQ-4 scores and eating disorder symptom scores (*p* ≤ 0.001). The strength of this association varies significantly between persons (*p* ≤ 0.001).

### 3.3. Moderator Analyses

Main and interaction effects obtained from the HLM models for age, mood dysregulation, self-esteem, and perfectionism are presented in Table 2 (models 2 to 5). Statistically significant main effects were found for all potential moderators. Increases in age, mood dysregulation, self-esteem, and perfectionism are associated with increased eating disorder symptom severity (all *p* ≤ 0.001). 

Statistically significant interaction effects were found for age and PHQ-4 scores, i.e., in younger participants, anxiety/depression and eating disorder symptoms seem to be more strongly associated (model 2, *p* < 0.05). No statistically significant moderation effects of mood dysregulation, self-esteem, and perfectionism were found (*p* > 0.05).

## 4. Discussion

Our goal was to examine moderators of the link between anxiety/depression and eating disorder-related impairment in female adolescents and young adults. Former studies mainly made comparisons between individuals with clinical eating disorders with and without comorbid anxiety disorder and depression. These studies used, in part, relatively small samples while focusing primarily on a few demographic characteristics and eating disorder-related aspects (e.g., age at onset, bingeing and vomiting) [27,28,29]. Similarly, a recent study that explored anxiety and depression symptomatology in adult eating disorder patients focused primarily on eating disorder subtype and symptoms, medical comorbidities, and sociodemographic causes [30]. The present study allowed testing moderator effects for the longitudinal within-person association of anxiety/depression and eating disorder-related impairment in a large at-risk sample using HLM. Findings provide insights into the factors that moderate anxiety/depression and eating disorder-related impairment and may pave the way for subsequent research.

In line with previous findings [11,15,16,21,25], greater anxiety/depression was associated with more severe eating disorder symptomatology. Thus, results support the cumulative detrimental effect of co-occurring anxiety, depression, and eating disorder symptoms. In total, 81% of the variance in eating disorder symptoms could be explained by differences on the between-person level. We hypothesized that age, mood dysregulation, self-esteem, and perfectionism could explain the variability found in the association of anxiety/depression and eating disorder symptomatology.

Results suggest greater eating disorder symptom severity in individuals reporting dysfunctional mood regulation, low self-esteem, and high levels of perfectionism, adding to the available evidence indicating that low self-esteem, high perfectionism, and dysfunctional emotion regulation are related to development and maintenance of eating disorders. In contrast to the hypothesis, moderating effects for mood dysregulation, self-esteem, and perfectionism appear to be negligible except for participants’ age.

Maladaptive emotion regulation is associated with greater vulnerability to more severe anxiety, depression, and eating disorder psychopathology [31,32]. Further, low self-esteem and clinical perfectionism present key maintaining and transdiagnostic factors in eating disorders [36]. Low self-esteem is a risk factor for development of depression in adolescents and young adults [37,42,43] and associated with anxiety disorder [44,45]. Elevated levels of perfectionism were linked to anxiety and depression in both adolescents with clinical and sub clinical eating disorders [49,50,51]. In conclusion, results show a relationship between each of the factors examined, i.e., mood dysregulation, self-esteem, and perfectionism, and eating disorder symptomatology, confirming prior research. However, the lack of moderation of effects of mood dysregulation, self-esteem, and perfectionism stands in contrast to assumptions and evidence from the literature.

Statistically significant moderation effects could only be demonstrated for participants’ age. Whereas late adolescence and young adulthood were linked to more severe eating disorder symptoms, young participants showed a stronger link between anxiety/depression and eating disorder symptomatology. While onset of a clinical eating disorder in itself peaks in middle to late adolescence, young females appear to be especially vulnerable to the co-occurrence of elevated sub-threshold anxiety, depression, and eating disorder symptomatology [69].

### 4.1. Implications

Several aspects are of interest for future research and practice. Analyses of specific eating disorder diagnoses or symptoms in relation to anxiety and depression, respectively, may be worthwhile as differences in prevalence rates for comorbidities were found [12,23]. Emotion regulation plays a role in onset and maintenance of depression and anxiety [70]. Theoretical models of eating disorders assume that maladaptive emotion regulation strategies such as disordered eating are endorsed to downregulate emotions, increasing the risk for threshold eating disorders [36,71]. A meta-analytic review emphasized the detrimental effect of the presence of maladaptive strategies in contrast to absence of adaptive strategies [33]. In further studies, the use of specific emotion-regulation strategies in relation to eating disorders, anxiety, and depression could be examined, as well as their role as potential risk and protective factors.

The present study examined self-esteem, perfectionism, and mood dysregulation as potential moderators of anxiety, depression, and eating disorders in young females. Two adaptations for future research come to mind, i.e., the inclusion of male participants and the examination of additional moderators.

The current sample consists of young females with elevated eating disorder risk, and thus one of the most studied groups in eating disorder research. Therefore, the inclusion of understudied groups, such as male adolescents and adults, seems desirable for future studies. Interest in male eating disorders has received a boost in recent years as prevalence rates have increased [72], emphasizing the need for the examination of anxiety, depression, and eating disorders also in male populations.

Regarding further moderators, studies may incorporate social aspects such as peers or family, social withdrawal, and feelings of loneliness. Social media and Internet use have emerged as mental health research area possibly affecting body image concerns and interaction with peers [73]. Education and socioeconomic status as well as familial mental disorders, trauma or adverse childhood experiences are associated with onset of several mental disorders and may be able to explain variability in the association between anxiety/depression and eating disorder symptomatology.

Substantial differences were found in the link between anxiety/depression and eating disorder symptomatology in young females. Apart from their age, reasons for the differences in anxiety/depression and eating disorder-related impairment remain unexplained. Still, all three disorders are associated with high prevalence rates and a high burden of disease, particularly in the case of the co-occurrence of anxiety disorders, depression, and eating disorders. Since young adolescents are particularly at risk, prevention efforts should focus at reaching young people early in order to be effective. Considering the significant impairment as well as the inconsistent evidence found, the deployment of more personalized, preventive interventions adaptable to individuals’ longitudinal course of symptoms related to anxiety, depression, and eating disorders may be most promising.

### 4.2. Limitations

Some limitations need to be mentioned. First, data collection was not specifically designed for the purpose of this study, i.e., for the examination of moderation effects on anxiety/depression and eating disorder-related impairment. For instance, future studies may benefit from using more widely used questionnaires. However, the self-developed eating disorder symptom questionnaire showed excellent psychometric properties and concordance with validated eating disorder measures, such as the EDE-Q and CR-EAT. The CR-EAT was answered voluntarily at the beginning of participation, resulting in fewer participants being included in the analyses of self-esteem, perfectionism, and mood dysregulation. Nevertheless, the sample size of 238 individuals was still sufficiently large for the conducted analyses. The generalizability of the findings may be limited to female adolescents and young adults.

In addition, sampling design enabled cross-sectional analyses only, which did not allow for inferences on the causality of the effects. By applying ecological momentary assessment (EMA), future studies may be able to analyze the time-deferred effects of anxiety/depression and eating disorder symptomatology as EMA comes with the benefits of real-time assessments in real-world settings with high ecological validity producing extensive data over short periods of time.

## 5. Conclusions

In line with previous findings, a strong association between anxiety/depression and eating-disorder-related impairment was found. Results further corroborate existing evidence that female adolescents and young adults with low self-esteem, high perfectionism, and dysfunctional mood regulation demonstrate more severe eating disorder symptoms. No moderating effects of self-esteem, perfectionism, and mood dysregulation regarding the link between anxiety, depression, and eating disorder symptomatology were found. Younger participants appeared as a specific risk group for impairment related to both anxiety/depression and eating disorder, which should be considered explicitly in prevention work.

## Figures and Tables

**Table 1:** Sample characteristics.

Scale		Total (*N* = 320)	
	Age, years	M (SD)	16.98 (3.61)
	BMI	M (SD)	20.32 (3.07)
WCS	Weight and shape concerns	M (SD)	56.65 (28.57)
	Eating disorder treatment	%	15.63
EDE-Q ^1^	Eating disorder psychopathology	M (SD)	2.87 (1.82)
SEED	Vomiting	%	25.00 ^2^
SEED	Laxative use	%	10.31 ^2^
SEED	Dieting	%	58.75 ^2^
SEED	Excessive exercising	%	50.31 ^2^
SEED	Bingeing	%	56.25 ^2^
PHQ-4 ^3^	Depression/Anxiety	M (SD)	4.80 (3.64)
CREAT	Mood Dysregulation	M (SD)	3.93 (1.50)
CREAT	Self-Esteem	M (SD)	3.56 (1.33)
CREAT	Perfectionism	M (SD)	3.78 (1.12)

Note. ^1^ Global eating disorder psychopathology. ^2^ Engaged in this behavior at least once in the past week. ^3^ Person-mean over all repeated measurements, with higher scores indicating probable presence of anxiety disorder or depression.

**Table 2:** Results of the HLM (maximum likelihood) models for the association of eating disorder symptomatology and anxiety/depression ^1^ and effects of moderators ^2^.

Model	1	2	3	4	5
	Anxiety/Depression	Age	Mood Dysregulation	Self-Esteem	Perfectionism
Fixed parameters					
Intercept	1.559 (0.066) ***	0.958 (0.117) ***	1.380 (0.088) ***	1.354 (0.090) ***	1.300 (0.109) ***
PHQ-4 cent.^3^	0.062 (0.006) ***	0.128 (0.029) ***	0.073 (0.026) **	0.035 (0.024)	0.086 (0.027) **
PHQ-4 mean^4^	0.161 (0.007) ***	0.150 (0.007) ***	0.121 (0.013) ***	0.120 (0.012) ***	0.138 (0.010) ***
Age		0.038 (0.007) ***			
PHQ-4 × Age		−0.004 (0.002) *			
Mood Dysregulation			0.106 (0.031) ***		
PHQ-4 × Mood Dysregulation			−0.003 (0.006)		
Self-Esteem				0.125 (0.033) ***	
PHQ-4 × Self-Esteem				0.007 (0.006)	
Perfectionism					0.106 (0.031) ***
PHQ-4 × Perfectionism					−0.006 (0.007)
					
Random parameters					
Residuum	0.095 (0.003) ***	0.095 (0.003) ***	0.090 (0.004) ***	0.090 (0.004) ***	0.090 (0.004) ***
Intercept	0.179 (0.016) ***	0.162 (0.015) ***	0.173 (0.019) ***	0.175 (0.020) ***	0.174 (0.019) ***
PHQ-4 cent. ^3^	0.003 (0.001) ***	0.002 (0.001) ***	0.003 (0.001) ***	0.003 (0.001) ***	0.003 (0.001) ***

Note. ^1^ Association between eating disorder symptomatology and anxiety/depression (Patient Health Questionnaire-4 (PHQ-4) scores) is shown in model 1. ^2^ Moderation effects for age, mood dysregulation, self-esteem, and perfectionism are shown in models 2 to 5, respectively. ^3^Person-mean-centered PHQ-4. ^4^PHQ-4 person mean. * *p* ≤ 0.05. ** *p* ≤ 0.01. *** *p* ≤ 0.000.

## Data Availability

The data presented in this study are available on request from the corresponding author. The data are not publicly available due to legal and privacy issues.
